# Muscle damage in oblique lumbar interbody fusion and unilateral biportal endoscopic-transforaminal lumbar interbody fusion for single-level lumbar degeneration diseases: retrospective cohort study

**DOI:** 10.1093/bjsopen/zraf182

**Published:** 2026-03-14

**Authors:** Tangyiheng Chen, Xin Wang, Renjie Li, Sen Yang, Xuefeng Li, Yi Zhu, Hong Zhou, Ying Zhuang, Han Sun, Weimin Jiang, Yijie Liu

**Affiliations:** Department of Orthopaedic Surgery, Fourth Affiliated Hospital of Soochow University (Dushu Lake Hospital Affiliated to Soochow University), Suzhou Medical College, Soochow University, Suzhou, Jiangsu, China; Department of Orthopaedic Surgery, Fourth Affiliated Hospital of Soochow University (Dushu Lake Hospital Affiliated to Soochow University), Suzhou Medical College, Soochow University, Suzhou, Jiangsu, China; Department of Orthopaedic Surgery, Fourth Affiliated Hospital of Soochow University (Dushu Lake Hospital Affiliated to Soochow University), Suzhou Medical College, Soochow University, Suzhou, Jiangsu, China; Department of Orthopaedic Surgery, Fourth Affiliated Hospital of Soochow University (Dushu Lake Hospital Affiliated to Soochow University), Suzhou Medical College, Soochow University, Suzhou, Jiangsu, China; Department of Orthopaedic Surgery, First Affiliated Hospital of Soochow University, Suzhou Medical College, Soochow University, Suzhou, Jiangsu, China; Department of Orthopaedic Surgery, Beijing Hospital, National Center of Gerontology, Beijing, China; Department of Orthopedics, Union Hospital, Tongji Medical College, Huazhong University of Science and Technology, Wuhan, China; Department of Orthopaedic Surgery, Wuxi Ninth People’s Hospital Affiliated to Soochow University, Wuxi, Jiangsu, China; Department of Articular Orthopaedics, The Third Affiliated Hospital of Soochow University, Changzhou, China; Department of Orthopaedic Surgery, Fourth Affiliated Hospital of Soochow University (Dushu Lake Hospital Affiliated to Soochow University), Suzhou Medical College, Soochow University, Suzhou, Jiangsu, China; Department of Orthopaedic Surgery, First Affiliated Hospital of Soochow University, Suzhou Medical College, Soochow University, Suzhou, Jiangsu, China; Department of Orthopaedic Surgery, Fourth Affiliated Hospital of Soochow University (Dushu Lake Hospital Affiliated to Soochow University), Suzhou Medical College, Soochow University, Suzhou, Jiangsu, China

**Keywords:** OLIF, UBE-TLIF, psoas muscles, paraspinal muscles, minimally invasive spine surgery

## Abstract

**Background:**

Iatrogenic injury and approach-related complications in lumbar fusion surgery have become significant concerns. This study aims to assess the muscle damage with oblique lumbar interbody fusion (OLIF) compared with unilateral biportal endoscopic-transforaminal lumbar interbody fusion (UBE-TLIF). This assessment will guide better clinical surgical choice in lumbar degeneration diseases.

**Methods:**

This was a retrospective study comparing OLIF combined with anterolateral single screw–rod fixation and UBE-TLIF combined with percutaneous pedicle screws under navigation from January 2021 to December 2023. Individuals with single-level degenerative lumbar disease participated in the study. This study compared the baseline and perioperative parameters (blood loss, operation time, and haematological indicators) and clinical outcome (visual analogue scale scores, Japanese Orthopaedic Association scores, fusion rate, and complications). Measurements of the total cross-sectional area of psoas muscles and paraspinal muscles were obtained using regions of interest defined by manual tracing.

**Results:**

The cohort comprised 192 patients: 112 underwent OLIF (64 female; mean age 65.1 years) and 80 underwent UBE-TLIF (44 female; mean age 64 years). The operation time (*P* < 0.001), blood loss (*P*  *<* 0.001), and creatine kinase (*P*  *<* 0.001) were significantly lower in the OLIF group than in the UBE-TLIF group. The visual analogue scale score of the back was lower in the OLIF group than in the UBE-TLIF group (*P*  *<* 0.001), whereas the visual analogue scale score of the leg was higher in the OLIF group than in the UBE-TLIF group (*P*  *<* 0.001). The changes in the total cross-sectional area in the psoas muscles were significantly higher in the OLIF group than in the UBE-TLIF group (*P*  *<* 0.001). The changes in the total cross-sectional area in the paraspinal muscles were significantly lower in the OLIF group than in the UBE-TLIF group (*P*  *<* 0.001). There were significant differences in the fatty infiltration of the paraspinal muscles between the two groups (*P*  *<* 0.001). The incidence of complications was comparable between the two groups (*P* = 0.874).

**Conclusion:**

With OLIF there were lower creatine kinase levels, less blood loss, and less damage to paraspinal muscles. Therefore, in cases where both procedures are equally appropriate, OLIF is safer and less damaging to muscles and is the first choice for patients.

## Teaser

Iatrogenic injury to the paraspinal muscles, disruption of the posterior tension band, and approach-related complications in lumbar fusion surgery have become significant concerns. The rapid advancement of endoscopic techniques over the past few decades has facilitated the application of spinal endoscopic methods to lumbar intervertebral fusion surgery. Oblique lumbar interbody fusion is becoming increasingly popular among spinal surgeons because of its minimally invasive nature.

## Introduction

Lumbar fusion surgery is a commonly employed procedure for various spinal ailments. Traditional open posterior approaches that necessitate extensive dissection of the paraspinal musculature can lead to permanent denervation of the erector spinae, functional loss, and late-onset spinal instability. Consequently, iatrogenic injury to the paraspinal muscles, disruption of the posterior tension band, and approach-related complications have become significant concerns. Recently, there has been increased interest in the role of paravertebral muscles as predictors of surgical outcomes in spinal procedures. Research indicates that the elevation and continuous wide retraction of paraspinal muscles can result in ischaemia, denervation, and dysfunction, which contribute to chronic pain and suboptimal postoperative clinical outcomes^1–3^. Tsutsumimoto *et al*.^4^ suggest that the sustained stretching of paraspinal muscles caused by retractors elevates the pressure within these muscles, adversely affecting the blood perfusion of their capillaries during traditional open surgery. This ischaemic change in paraspinal muscles ultimately leads to functional alterations and muscle atrophy. Alarmingly, statistics reveal that approximately 30% of patients experience exacerbated back pain after surgery compared with their preoperative status^5^. Thus, iatrogenic muscle injury has emerged as a major concern for practising spine surgeons.

Several studies emphasize the prevalence of invasive muscle damage associated with posterior spinal surgeries. For instance, the traditional procedure of posterior lumbar interbody fusion has been demonstrated to lead to increased degeneration of the multifidus muscle and heightened postoperative low back pain when contrasted with minimally invasive methods like percutaneous pedicle screw fixation^6,7^. Consequently, minimally invasive spine surgery has garnered increasing attention in recent years. The rapid advancement of endoscopic techniques over the past few decades has facilitated the application of spinal endoscopic methods to lumbar intervertebral fusion surgery. Recently, unilateral biportal endoscopic-transforaminal lumbar interbody fusion (UBE-TLIF) has emerged as a notable technique, as it features independent endoscopic and instrument channels. Furthermore, the efficacy and validity of minimally invasive lumbar interbody fusions utilizing percutaneous pedicle screws for spondylodiscitis have been documented^8,9^. Clinical trials^10^ indicate that these minimally invasive interbody fusions effectively minimize muscle injuries. In addition, oblique lumbar interbody fusion (OLIF) is becoming increasingly popular among spinal surgeons because of its minimally invasive nature. This method is specifically aimed at the anterolateral region of the intervertebral disc located between the psoas major and major vasculature through the oblique lateral corridor. Additionally, the Wiltse technique reduces damage to the posterior paravertebral muscles, which in turn lowers the likelihood of experiencing chronic low back pain following surgery^11,12^. Additionally, OLIF facilitates correction of spondylolisthesis and rotatory deformities, as well as indirect nerve decompression through ligamentotaxis. These benefits may lead to reduced surgical pain and expedited recovery compared with traditional surgical methods^13^.

The advantages of minimally invasive techniques over open techniques include reduced blood loss, less surgical site pain, faster recovery, and a lower incidence of postoperative wound infections. The aim of this study was to assess the muscle changes at the operated level in OLIF compared with UBE-TLIF. This assessment will provide insight into the potential disability patients may experience during their postoperative period. To evaluate the invasiveness and tolerability of UBE-TLIF in comparison with OLIF, this study assessed serum markers of muscle damage and inflammation, conducted quantitative analyses using magnetic resonance imaging (MRI), and measured surgical pain, blood loss, and postoperative recovery of activities.

## Methods

### Basic demographics and serology

This study was conducted at six participating centres in China. The study duration was from January 2021 to December 2023. Ethical approval was received from the Institutional Ethics committee, reference number 211046. This is a retrospective study. Individuals with single-level degenerative lumbar disease participated in the study. Before the operation, patients were told the disadvantages and advantages of the two procedures. They could choose the procedure themselves. Informed consent was obtained from those who agreed to allow the use of clinical data, including imaging. Routine follow-up was performed at 3 days, 1 month, 3 months, 6 months, 1 year after surgery, and then every 6 months thereafter. All patients were followed up for at least 24 months. If one patient was followed up for two years, then the final follow-up was 2 years. Exclusions from the study included patients undergoing revision lumbar surgery, those who had decompression-only procedures, individuals with multilevel fusion surgeries, those with diagnoses unrelated to degenerative conditions (such as infection, tumours, and so on), or those with a previous history of radiation treatment.

Demographic data for both groups were gathered before surgery, and included age, sex, body mass index (BMI), diagnosis, and the specific surgical level. Information related to the perioperative period, such as surgical duration, blood loss, and haematological markers, was documented both before and after the operation. A strobe checklist was added in the supplementary template.

### Clinical and radiological indicators

**Fig. 1:**
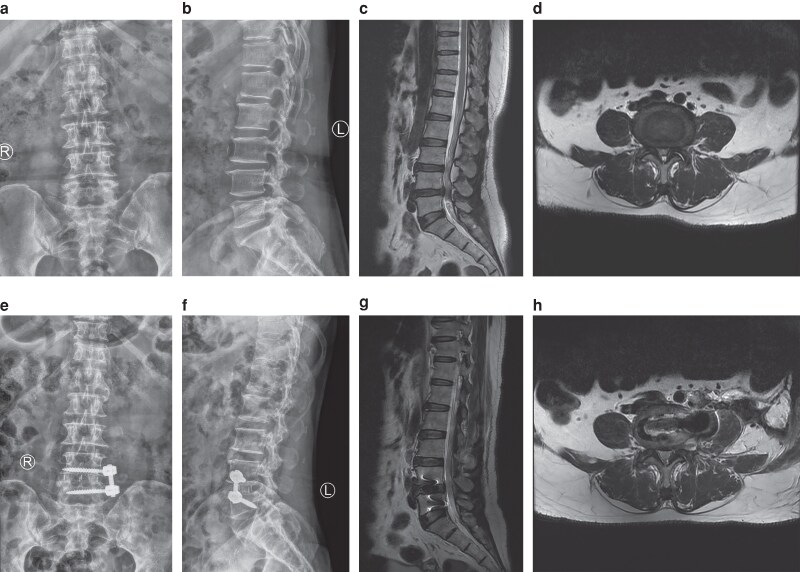
A 63-year-old female with lumbar disc herniation who underwent L4-5 oblique lumbar interbody fusion **a, b** preoperative anterolateral X-ray; **c** preoperative T2 sagittal MRI showed L4-5 herniation; **d** preoperative T2 axial MRI; **e, f** postoperative anterolateral X-ray; **g** preoperative T2 sagittal MRI; **h** preoperative T2 axial MRI. MRI, magnetic resonance imaging.

**Fig. 2:**
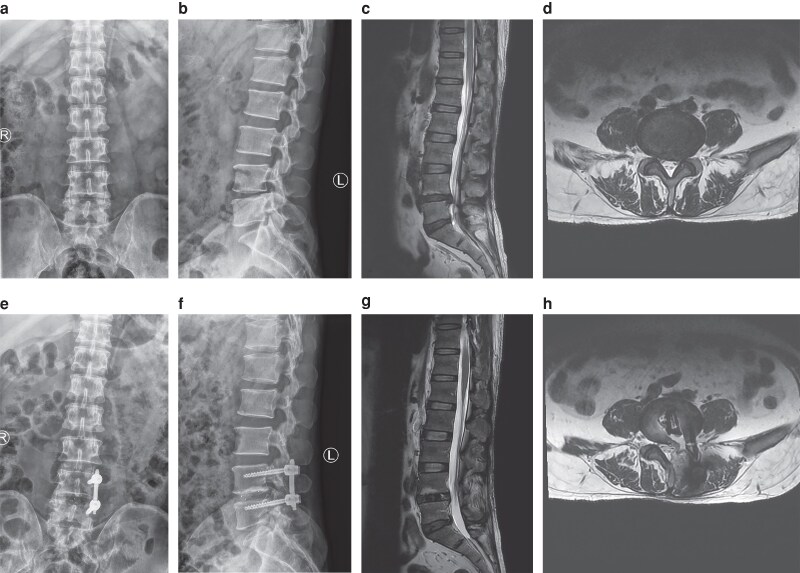
A 56-year-old male with lumbar disc herniation who underwent L4-5 unilateral biportal endoscopic-transforaminal lumbar interbody fusion **a, b** preoperative anterolateral X-ray; **c** preoperative T2 sagittal MRI showed L4-5 herniation; **d** preoperative T2 axial MRI; **e, f** postoperative anterolateral X-ray; **g** preoperative T2 sagittal MRI; **h** preoperative T2 axial MRI. MRI, magnetic resonance imaging.

The baseline health status of patients was assessed both before and after surgery. Japanese Orthopaedic Association (JOA) and visual analogue scale (VAS) scores were documented before surgery as well as at 3 days, 1 month, 3 months, and 12 months following the operation. The fusion rate was evaluated using Bridwell’s fusion grading system^11^ at the 3-month, 6-month, and final follow-up periods. Spinal fusion was categorized as grades I and II. Two independent observers conducted all radiographic assessments, and the average of their measurements was utilized for analysis. Additionally, any perioperative complications were noted. The typical cases are shown in *Fig. 1* and *Fig. 2*.

**Fig. 3:**
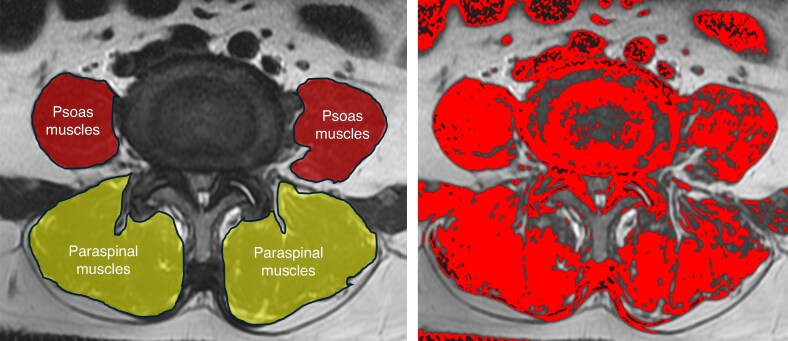
Bilateral manual segmentation of psoas and posterior paraspinal muscles on preoperative axial T2-weighted magnetic resonance imaging Cross-sectional area measured by Image J.

Muscle assessments were performed by trained research staff on standard axial T2-weighted MRI, specifically targeting the mid-disk space for both psoas and paraspinal muscles using Image J (*Fig. 3*). A spine surgeon and two clinical researchers received comprehensive training in the Goutallier classification system^14^, with cases assigned randomly without previous knowledge of the specific procedure. The muscle grading criteria were as follows: grade 0 indicates no intramuscular fat; grade 1 indicates the presence of minor fatty streaks; grade 2 shows fat that is apparent but still less than the muscle tissue; grade 3 signifies an equal amount of fat and muscle; and grade 4 denotes a higher fat content compared with muscle tissue.

### Statistical analysis

Analyses of statistical data were conducted using SPSS^®^ Statistics version 26.0 (IBM, Armonk, NY, USA). For parametric data, results are reported as mean(standard deviation(s.d.)) and analysed using the Student *t*-test. Nonparametric data are expressed as median (i.q.r.) and are evaluated with the Mann-Whitney U-test. Nominal data were analysed using the chi-square test. *P-*values of less than 0.05 were deemed statistically significant.

## Results

This retrospective study enrolled 192 patients: 112 OLIF with lateral vertebral screws and 80 UBE-TLIF with percutaneous pedicle screws under navigation. Patient demographics and preoperative characteristics are summarized in *Table 1*. There were no statistical differences in the age, sex, BMI, lesion level, and preoperative diagnoses between patients undergoing OLIF and UBE-TLIF. The operation time of OLIF was shorter than UBE-TLIF (89.9 ± 13.0 minutes (min) *versus* 121.5 ± 14.7 min; *P*  *<* 0.001), and the blood loss with OLIF was less than with UBE-TLIF (29.8 ± 9.9 ml *versus* 59.0 ± 24.7 ml; *P*  *<* 0.001).

**Table 1:** Patient demographics and preoperative characteristics

	OLIF (*n* = 112)	UBE-TLIF (*n* = 80)	P*
**Sex**			0.883
Male	48 (42.9%)	36 (45.0%)	
Female	64 (57.1%)	44 (55.0%)	
Age (years), mean(s.d.)	65.1(5.4)	64.6(6.6)	0.601
BMI (kg/m^2^), mean(s.d.)	24.2(2.7)	24.1(2.6)	0.786
**Diagnosis**			0.957
Lumbar spinal canal stenosis	80 (71.4%)	56 (70.0%)	
Lumbar disc herniation	32 (28.6%)	24 (30.0%)	
**Lesion level**			0.701
L3-4	32 (28.6%)	20 (25.0%)	
L4-5	80 (71.4%)	60 (75.0%)	
Operation time (min), mean(s.d.)	89.9(13.0)	121.5(14.7)	< 0.001
Blood loss (ml), mean(s.d.)	29.8(9.9)	59.0(24.7)	< 0.001

Values are *n* (%) unless otherwise stated. OLIF, oblique lumbar interbody fusion; UBE-TLIF, unilateral biportal endoscopic-transforaminal lumbar interbody fusion; s.d., standard deviation; BMI, body mass index.*χ2 test or Student t-test.

Comparison of follow-up clinical outcomes are outlined in *Table 2*. The mean(s.d.) VAS scores decreased, and the corresponding JOA scores increased in both groups. The VAS scores of the back with OLIF were lower than those with UBE-TLIF at 3 days after surgery, whereas the VAS scores of the leg with OLIF were higher than those with UBE-TLIF. At 1 month, 3 months, and 12 months, there were no significant differences between two groups. In the OLIF group, using Bridwell’s fusion grading system^11^, there were 76 grade I and 36 grade II. In the UBE-TLIF group, there were 60 grade I and 20 grade II. The fusion rates did not differ between the two groups (*P* = 0.362).

**Table 2:** Comparison of follow-up outcomes

	OLIF (*n* = 112)	UBE-TLIF (*n* = 80)	P*
**VAS (back)**			
Preoperative, mean(s.d.)	8.3(1.4)	8.4(1.5)	0.710
Postoperative 3 days, mean(s.d.)	3.1(1.0)	4.3(1.4)	< 0.001
1 month, mean(s.d.)	1.6(1.0)	1.9(1.3)	0.108
3 months, mean(s.d.)	1.6(0.9)	1.7(1.1)	0.592
12 months, mean(s.d.)	1.5(0.9)	1.5(1.0)	1.000
**VAS (leg)**			
Preoperative, mean(s.d.)	8.5(1.3)	8.4(1.4)	0.340
Postoperative 3 days, mean(s.d.)	4.0(1.2)	3.0(0.9)	< 0.001
1 month, mean(s.d.)	1.9(0.8)	1.8(1.0)	0.347
3 months, mean(s.d.)	1.8(0.9)	1.7 (1.0)	0.383
12 months, mean(s.d.)	1.6(0.9)	1.6(1.1)	0.777
**JOA score**			
Preoperative, mean(s.d.)	8.2(1.0)	8.0(1.0)	0.142
Postoperative 3 days, mean(s.d.)	13.0(1.2)	13.1(1.0)	0.545
1 month, mean(s.d.)	15.3(1.1)	15.3(1.3)	0.959
3 months, mean(s.d.)	15.5(0.9)	15.6(1.1)	0.597
12 months, mean(s.d.)	15.7(0.8)	15.9(1.0)	0.086
**Fusion rate**			0.362
Grade I	76 (67.9%)	60 (75.0%)	
Grade II	36 (32.1%)	20 (25.0%)	
Grade III	0 (0.0%)	0 (0.0%)	
Grade IV	0 (0.0%)	0 (0.0%)	
**Complications**	17.8%	18.7%	0.874
Spinal cord hypertension syndrome	0 (0.0%)	8 (10.0%)	
Dural injury	0 (0.0%)	4 (5.0%)	
Hip flexion weakness	12 (10.7%)	0 (0.0%)	
Cage subsidence	8 (7.1%)	3 (3.7%)	
Vascular or peritoneal injury	0 (0.0%)	0 (0.0%)	

Values are *n* (%) unless otherwise stated. OLIF, oblique lumbar interbody fusion; UBE-TLIF, unilateral biportal endoscopic-transforaminal lumbar interbody fusion; VAS, visual analogue scale; s.d., standard deviation; JOA, Japanese Orthopaedic Association. *χ2 test or Student t-test.

There was no significant difference between the OLIF group and the UBE-TLIF group with regards to the rate of complications (17.8% *versus* 18.7%; *P* = 0.874). Specifically, in the OLIF group, 12 patients experienced hip flexion weakness. The symptoms completely disappeared after relieving swelling treatment and routine guidance of functional exercise. Eight patients experienced cage subsidence; however, they achieved good fusion with osteogenesis therapy in the last follow-up. In the UBE-TLIF group, eight patients experienced spinal cord hypertension syndrome. The symptoms completely disappeared after sedation and hormone treatment. Four patients experienced dural injury. They recovered well after treatment such as reducing stress and anti-infective therapy. Three patients experienced cage subsidence. They achieved good fusion in the last follow-up. There was no vascular or peritoneal injury in either group.

Blood and biochemical tests are shown in *Table 3*. There were no significant differences in the levels of albumin and prealbumin before and after surgery, whereas the differences were significant for hematocrit, haemoglobin, and red blood cell levels. C-reactive protein (CRP) and white blood cell (WBC) levels increased after surgery. However, there were no significant differences between OLIF and UBE-TLIF. Postoperative creatine kinase (CK) increased significantly with UBE-TLIF compared with OLIF (mean(s.d) 421.6(220.9) U/L *versus* 176.3(124.5) U/L; *P*  *<* 0.001).

**Table 3:** Comparison of perioperative condition between two groups

	OLIF (*n* = 112)	UBE-TLIF (*n* = 80)	P*
Preoperative HCT (%)	39.9(2.4)	39.5(2.3)	0.367
Postoperative HCT (%)	37.6(3.4)	35.4(3.1)	< 0.001
Preoperative Hb (g/L)	135.3(8.7)	135.1(8.4)	0.882
Postoperative Hb (g/L)	127.9(10.3)	122.5(10.7)	< 0.001
Preoperative RBC	4.3(0.3)	4.3(0.3)	0.893
Postoperative RBC	4.1(0.4)	3.9(0.3)	< 0.001
Preoperative albumin	42.6(3.2)	42.7(2.1)	0.670
Postoperative albumin	38.9(2.7)	38.8(2.0)	0.862
Preoperative prealbumin	239.7.2(51.3)	232.0(51.5)	0.306
Postoperative prealbumin	215.9.9(45.9)	208.2(48.6)	0.268
Preoperative CK	144.8(94.7)	151.4(93.2)	0.783
Postoperative CK	176.3(124.5)	421.6(220.9)	< 0.001
Postoperative CRP	17.9(10.0)	18.0(7.0)	0.917
Postoperative WBC	13.4(1.8)	13.4(2.3)	0.783

Values are mean(standard deviation). OLIF, oblique lumbar interbody fusion; UBE-TLIF, unilateral biportal endoscopic-transforaminal lumbar interbody fusion; HCT: haematocrit; Hb, haemoglobin; RBC, red blood cell; CK, creatine kinase; CRP, C-reactive protein; WBC, white blood cell. *χ2 test or Student t-test.

The changes in TCSA in the psoas muscles were significantly higher in the OLIF group than in the UBE-TLIF group (mean(s.d.) 145.4(89.4) mm^2^  *versus* 95.2(33.4) mm^2^; *P*  *<* 0.001). The changes in the total cross-sectional area (TCSA) in the paraspinal muscles were significantly lower in the OLIF group than in the UBE-TLIF group (mean(s.d.) 55.4(208.4) mm^2^  *versus* 311.1(145.0) mm^2^; *P*  *<* 0.001). According to Goutallier classification, there were 0 grade 0, 29 grade 1, 55 grade 2, 21 grade 3 and 7 grade 4 for the psoas muscles in the OLIF group; 0 grade 0, 16 grade 1, 40 grade 2, 21 grade 3, and 3 grade 4 for the psoas muscles in the UBE-TLIF group; 20 grade 0, 72 grade 1, 20 grade 2, 0 grade 3, and 0 grade 4 for the paraspinal muscles in the OLIF group; and 0 grade 0, 8 grade 1, 32 grade 2, 36 grade 3, and 4 grade 4 for the paraspinal muscles in the UBE-TLIF group. There were significant differences in the fatty infiltration of paraspinal muscles between the two groups (*Table 4*).

**Table 4:** Muscle parameters before and after operations according to spinal level

	OLIF (*n* = 112)	UBE-TLIF (*n* = 80)	P*
**Psoas muscles**			
Preoperative TCSA (mm^2^), mean(s.d.)	976.8(209.3)	1022.0(171.3)	0.114
Postoperative TCSA (mm^2^), mean(s.d.)	1122.1(248.6)	1117.2(167.4)	0.869
Change in TCAS (mm^2^), mean(s.d.)	145.4(89.4)	95.2(33.4)	< 0.001
Goutallier classification			0.483
Grade 0	0 (0.0%)	0 (0.0%)	
Grade 1	29 (25.9%)	16 (20.0%)	
Grade 2	55 (49.1%)	40 (50.0%)	
Grade 3	21 (18.7%)	21 (26.3%)	
Grade 4	7 (6.3%)	3 (3.7%)	
**Paraspinal muscles**			
Preoperative TCSA (mm^2^), mean(s.d.)	1855.3(271.5)	1822.0(203.3)	0.333
Postoperative TCSA (mm^2^), mean(s.d.)	1910.6(289.7)	2133.0(247.6)	< 0.001
Change in TCAS (mm^2^), mean(s.d.)	55.3(208.4)	311.1(145.0)	< 0.001
Goutallier classification			< 0.001
Grade 0	20 (17.9%)	0 (0.0%)	
Grade 1	72 (64.3%)	8 (10.0%)	
Grade 2	20 (17.8%)	32 (40.0%)	
Grade 3	0 (0.0%)	36 (45.0%)	
Grade 4	0 (0.0%)	4 (5.0%)	

Values are *n* (%) unless otherwise stated. OLIF, oblique lumbar interbody fusion; UBE-TLIF, unilateral biportal endoscopic-transforaminal lumbar interbody fusion; TCSA, total cross-sectional area. *χ2 test or Student t-test.

## Discussion

Minimally invasive spine surgery was developed as a potential solution to reduce the incidence of iatrogenic soft-tissue injury while still achieving the traditional goals of open procedures. Two typical surgical techniques are OLIF and UBE-TLIF. OLIF employs a retroperitoneal fat and lumbar major muscle gap approach, effectively avoiding critical neurovascular structures, and is therefore gradually gaining acceptance among spine surgeons. The UBE-TLIF technique establishes its working area within the trigone of the multifidus muscle, utilizing the potential gap between the multifidus and the spinous process of the posterior lamina^15^. UBE-TLIF allows for the identification and complete removal of cartilage endplates under direct endoscopic visualization, creating an optimal environment for intervertebral fusion. The operation time of the UBE-TLIF group remained significantly longer than that of the OLIF group in this study. Such delicate endoscopic manipulation not only necessitates surgical experience but may also be time-consuming, potentially explaining why the UBE-TLIF group experienced longer surgical durations compared with OLIF. There were no severe complications requiring revision in either group. The different complications in the two groups were determined by the different characteristics of the two approaches. Specifically, because of the hydraulic pressure, cases of spinal cord hypertension syndrome occurred after UBE-TLIF. However, the symptoms completely disappeared after sedation and hormone treatment. Because of the irritation of the psoas muscle, hip flexion weakness occurred after OLIF. However, the symptoms completely disappeared after relieving swelling treatment and routine guidance of functional exercise. All the complications were temporary or disappeared after appropriate treatment. This indicates that both techniques are safe and effective.

In this study, both VAS scores and JOA scores showed significant improvement in both groups at each time point when compared with preoperative measurements. The clinical outcomes at 1 month, 3 months, and 1 year were comparable between the groups, with no significant differences observed. This suggests that both surgical techniques are advantageous for patients with lumbar degenerative disease, yielding similar medium- and short-term clinical results. Notably, for VAS scores related to back pain, the OLIF group demonstrated superior outcomes compared with the UBE-TLIF group at 3 days after surgery. This advantage can be attributed to the OLIF technique's preservation of the posterior spinal muscles and avoidance of bony structures such as the laminae, which minimizes peripheral tissue damage and reduces the risk of medically induced neurological disorders^16^. Conversely, for VAS scores concerning leg pain, the UBE-TLIF group outperformed the OLIF group at 3 days after surgery, likely due to the irritation of the psoas muscles caused by anterolateral single screw-rod fixation, which can lead to hip flexion weakness.

The use of serum markers for inflammation and muscle damage provides objective measures of the invasiveness of surgical procedures. Creatine kinase (CK) acts as a quantitative measure of muscle injury, and a relationship is noted between CK levels and the pressure applied to paraspinal muscles during retraction^17,18^. In a prospective cohort investigation, Arts *et al*.^19^ identified a dose–response relationship linking CK levels and the extent of surgical invasiveness. Muscle injury associated with spinal surgery leads to a rise in serum CK concentration, which is commonly employed for assessing muscle injury in the immediate postoperative period. In the authors’ research, both CRP and WBC levels showed an increase following surgery; however, significant differences were not observed between the OLIF group and the UBE-TLIF group. Kawaguchi *et al*.^17,20^ determined that back muscle injury, as indicated by elevated serum creatine phosphokinase levels, is directly related to retraction pressure, duration, and the extent of surgical exposure. Notably, postoperative CK levels were significantly higher in UBE-TLIF compared with OLIF. The findings of the authors of the present study suggest that, whereas both procedures improve various clinical outcome measures, OLIF significantly reduces paraspinal muscle injury relative to UBE-TLIF, as evidenced by less blood loss, lower serum CK levels, and a reduced incidence of postoperative low back pain.

In this retrospective study, the quantitative changes were examined in the occurring morphology of the psoas and paraspinal muscles. Kang *et al*.^21^ confirmed through MRI that the degree of paraspinal muscle degeneration can be assessed by the decrease in paraspinal muscle CSA and fatty infiltration. The changes in TCSA of the psoas muscles were significantly greater in the OLIF group compared with the UBE-TLIF group. Conversely, the changes in TCSA of the paraspinal muscles were significantly lower in the OLIF group than in the UBE-TLIF group. Notably, there were significant differences in the fatty infiltration of paraspinal muscles between the two groups. Previous studies^22^ have indicated that the reduction in muscle volume and the increase in fat deposition are primary characteristics of paraspinal muscle degeneration. In an animal model, the duration of retraction on the multifidus muscle was identified as a critical factor contributing to muscle injury and atrophy, with oedema, necrosis, and inflammation primarily occurring in the early stages, whereas fatty degeneration became pronounced 12 weeks after surgery and beyond^23^. This phenomenon aligns with the findings of this study. The fatty infiltration of the lumbar multifidus muscles following posterior surgeries is clinically associated with low back pain, which may explain why several patients experienced back pain after undergoing UBE-TLIF. Paraspinal muscle dysfunction, resulting from degeneration in the postoperative period, plays a crucial role in the progression to failed back syndrome. The utilization of UBE involves two notches to create passage channels (one for observation and another for operation) along with the ion-knife separation of portions of the paravertebral muscle and lamina attachment points, which results in artificially created cavities. Concurrently, the procedure continues to utilize high-pressure saline for irrigation. The repeated insertion and removal of instruments leads to greater injury to the paravertebral muscles. Kambin *et al*.^24^ conducted a follow-up study on 88 patients with lumbar degenerative diseases who underwent UBE surgery, assessing the extent of paravertebral muscle injury via MRI. Their findings indicated that the degree of postoperative ipsilateral paravertebral muscle injury increased with prolonged operation time, consequently extending recovery periods. Regarding the psoas muscles, the radiological assessment of the postoperative changes in the psoas major by the authors of the present study indicated that the TCSA on the approach side significantly increased after surgery, with a notable rise in the mean TCSA ratio in the OLIF group. They hypothesize that this condition may be attributed to temporary postoperative swelling resulting from intraoperative muscle retraction, suggesting that the OLIF approach minimally impacts the psoas major. Furthermore, this study considered that swelling of the psoas major or haematoma formation could have occurred due to surgical manipulation and irritation of the screw tail cap. Several patients experienced transient clinical weakness of hip flexion. Radiologically, some degree of atrophy was observed, likely due to compromised microcirculation in the psoas muscle; however, spontaneous recovery was noted in the later stages.

Some limitations of the present study warrant discussion. The hip flexor muscles include the iliopsoas, pectineus, rectus femoris, and adductor longus. This study assessed hip flexion weakness as an indicator of psoas muscle injury. However, isolating the specific muscle action proved challenging. Furthermore, this study did not conduct a histopathological assessment of the muscle, relying solely on MRI to evaluate muscle degeneration.

In conclusion, both OLIF and UBE-TLIF yield favourable outcomes in the management of single-level degenerative lumbar diseases. After surgery, OLIF was associated with temporary swelling of the psoas muscle; however, long-term damage to the psoas was not significant, and there was no evident damage to the erector spinae and multifidus muscles. In comparison with UBE-TLIF, OLIF demonstrated lower creatine kinase levels, reduced blood loss, and less muscle damage. Consequently, in cases where both procedures are equally indicated, OLIF is advocated as the safer and less-invasive option, making it the preferred choice for patients.

## Data Availability

Data are available from the corresponding author upon reasonable request. The data are not publicly available as this could compromise the privacy of research participants.
